# Patient-derived organoid facilitating personalized medicine in non-small cell lung cancer: two case reports

**DOI:** 10.3389/fonc.2025.1674897

**Published:** 2025-10-03

**Authors:** Lili Qin, Shasha Wang, Liping Li, Haifeng Qin

**Affiliations:** Department of Thoracic Oncology & Oncology Immunotherapy, Beijing GoBroad Hospital, Beijing, China

**Keywords:** case report, non-small cell lung cancer, organoid, brain metastasis, personalized medicine

## Abstract

Patients with brain metastases from lung cancer exhibit rapid disease progression and a poor prognosis, underscoring an urgent need for effective therapeutic strategies. Drug sensitivity testing using patient-derived organoids (PDOs) has emerged as a promising tool for guiding clinical treatment decisions. Here, we report two cases of non-small cell lung cancer (NSCLC) with brain metastases where treatment guided by PDO-based drug sensitivity screening aided in disease control. Case 1 involved a patient with an EGFR exon 19 deletion. The corresponding PDO model demonstrated sensitivity to a combination of pemetrexed, carboplatin, and osimertinib, but insensitivity to osimertinib monotherapy. Following this guidance, the patient achieved a partial response (PR) to the triplet regimen and was subsequently de-escalated to maintenance therapy. The patient’s disease remained stable at the time of this report. Case 2 involved a patient with a complex EML4-ALK fusion variant 3 (E6:A20) and a novel NRXN1-ALK fusion (N19:A20). The patient had progressed on multiple lines of therapy, including alectinib and lorlatinib. The PDO model showed sensitivity to brigatinib but insensitivity to ensartinib. Subsequent treatment with brigatinib induced a PR that was sustained for 5.8 months; the patient survived for a total of 9 months following the initiation of this PDO-guided therapy. These two cases suggests that PDOs derived from primary and metastatic lesions may help optimize treatment regimens for patients with lung cancer brain metastases, thereby enabling personalized therapy and potentially improving survival outcomes.

## Introduction

A 2022 survey documented 1.06 million new cases of lung cancer and 730,000 related deaths annually in China (1). The presence of brain metastases (BM) is strongly associated with severe morbidity and limited survival in patients with lung cancer (2). Data from the National Cancer Data Base (NCDB) reveal an overall incidence of BM of approximately 10% at initial diagnosis, rising to 26% in patients with stage IV disease and up to 40% during disease progression, with a median survival time of only 6 months (3). Approximately 70% of patients diagnosed with non-small cell lung cancer (NSCLC) harboring epidermal growth factor receptor (*EGFR*) mutations eventually develop brain metastases (4).

The phase III FLAURA2 study (NCT04035486) established that first-line osimertinib combined with platinum-pemetrexed chemotherapy has a manageable safety profile in metastatic EGFR-mutated NSCLC (5). Results from this trial confirmed superior progression-free survival (PFS) in the osimertinib-chemotherapy group (median PFS 24.0 months) versus the osimertinib monotherapy group (median PFS 15.3 months) (6). However, in clinical practice, patients achieving an objective response often desire a reduction in treatment burden during maintenance. Such a de-escalation requires rigorous, evidence-based assessment to avoid compromising efficacy.

Among patients with anaplastic lymphoma kinase (ALK)-rearranged NSCLC, BM is present in 23.8% at the time of advanced disease diagnosis (7). The majority (85%) of these are EML4-ALK variants (8). The EML4-ALK fusion variant 3 (v3), resulting from a fusion between exon 6 of EML4 and exon 20 of ALK (E6:A20), is associated with greater resistance to second- and third-generation ALK inhibitors compared to first-generation agents (9). Consequently, data are lacking to inform treatment selection for patients with the ALK v3 fusion, particularly when co-occurring with novel fusions after progression on standard therapies.

Patient-derived organoids (PDOs) are three-dimensional *in vitro* models generated from a patient’s tumor tissue, which are increasingly used for predicting therapeutic response and facilitating personalized medicine (10). Here, we describe two cases of NSCLC with brain metastases where PDO-based drug sensitivity screening helped guide therapy, leading to objective clinical responses.

## Case report

### Case 1

In mid-December 2022, a 52-year-old male presented with a headache, dizziness, blurred vision, intermittent nausea, and a “walking on cotton” sensation in his feet. On January 13, 2023, a cranial MRI revealed a malignant tumor in the right cerebellopontine angle (CPA), with significant surrounding edema. The patient underwent resection of the CPA tumor on January 17, 2023, which alleviated his symptoms. Pathological analysis confirmed metastatic pulmonary adenocarcinoma. A PET-CT scan on January 28, 2023, identified a hypermetabolic mass in the right lung with associated mediastinal lymphadenopathy.

**Figure 1:**
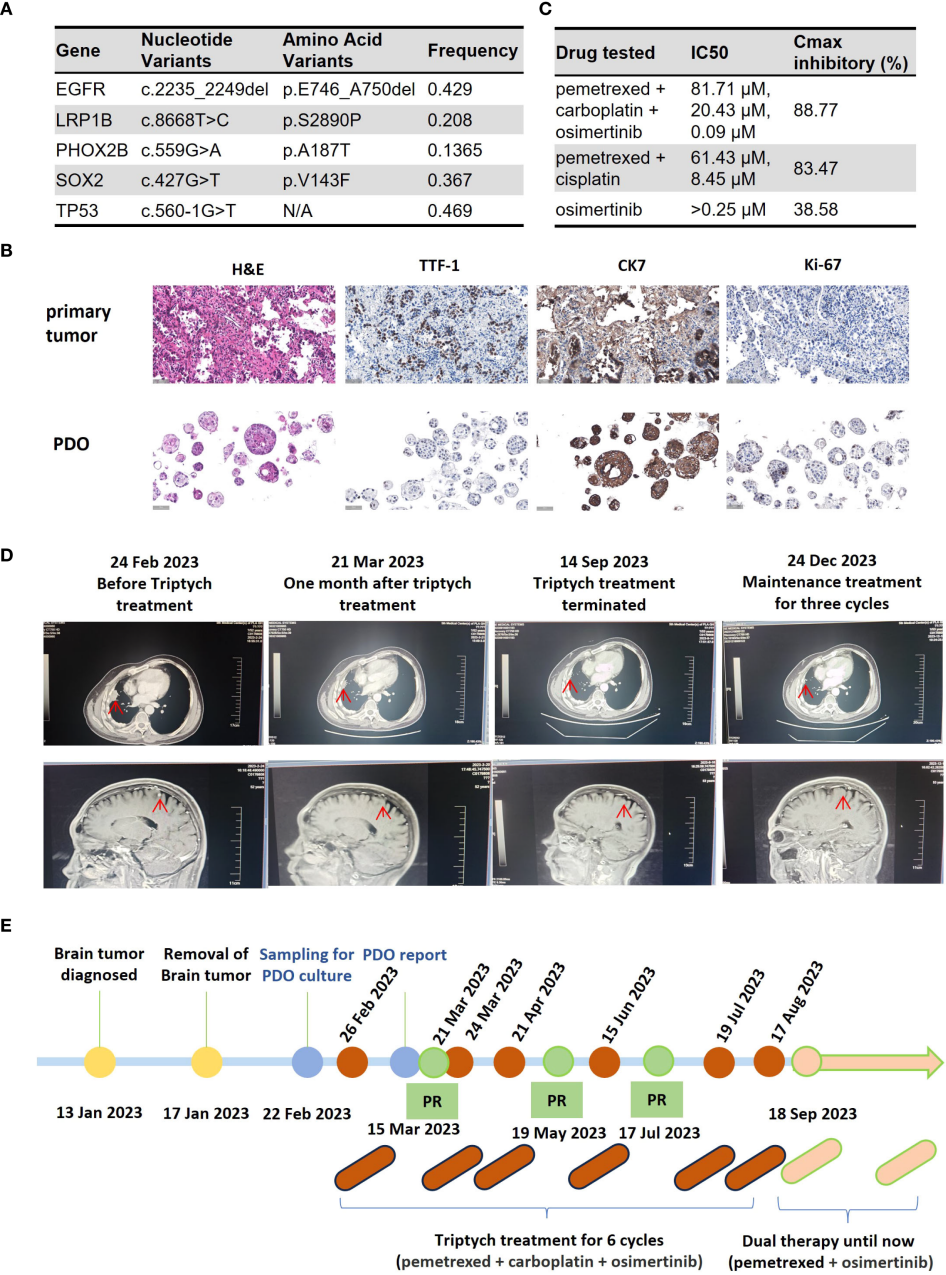
Clinical course of Case 1, a patient with *EGFR*-mutant NSCLC with brain metastases. **(A)** Table of somatic mutations detected in the tumor tissue sample. **(B)** Histological comparison confirming the fidelity of the patient-derived organoid (PDO) to the primary tumor via H&E and IHC staining for key biomarkers. **(C)** Results of the PDO drug sensitivity assay. Cell viability was measured using an ATP-based assay following drug exposure. The table displays the half-maximal inhibitory concentration (IC_50_) and the maximal inhibitory rate for each agent. (Data are presented as the mean of technical replicates; error bars and statistical tests were not reported). **(D)** Serial imaging confirming a partial response (PR) to treatment. **(E)** Timeline of the patient’s clinical course, from diagnosis through treatment and follow-up. (H&E, hematoxylin and eosin; IHC, immunohistochemistry; TTF-1, thyroid transcription factor-1; CK7, cytokeratin 7; PDO, patient-derived organoid; IC_50_, half-maximal inhibitory concentration).

On February 22, 2023, the patient underwent a CT-guided lung biopsy at our hospital for PDO culture and drug sensitivity testing (Beijing Daxiang Biotech, China). Pathological and genetic analyses confirmed lung adenocarcinoma with an EGFR exon 19 deletion and a *TP53* mutation (**Figure 1A**). While awaiting PDO results, the patient commenced empirical treatment on February 26, 2023, with a triplet regimen of pemetrexed (900 mg), carboplatin (500 mg), and osimertinib (80 mg orally). The PDO drug sensitivity report was issued on March 15, 2023. The results prospectively validated the choice of therapy, showing high sensitivity to the triplet combination (maximal inhibition: 88.77%) and the chemotherapy doublet (pemetrexed+cisplatin, 83.47%), but insensitivity to osimertinib monotherapy (38.58%) (**Figure 1C**). The PDO was histologically consistent with the source tumor (**Figure 1B**).

Imaging assessments confirmed a partial response (PR) (**Figure 1D**). On September 18, 2023, treatment was de-escalated to maintenance therapy with pemetrexed and osimertinib. The patient’s disease remained stable for over 8 months at the time of submission. The treatment timeline is shown in **Figure 1E**.

### Case 2

A 33-year-old male presented with pain and reduced mobility in the left lower limb. PET-CT on September 30, 2021, at an external hospital revealed lung cancer in the right lower lobe with obstructive atelectasis, multiple lymph node metastases (right supraclavicular, mediastinal, retroperitoneal), and bone metastases. Bronchoscopic pathology confirmed poorly differentiated adenocarcinoma. Genetic testing identified an EML4-ALK fusion (E6:A20) and a novel NRXN1-ALK fusion (N19:A20).

From October 2021 to June 2022, first-line alectinib yielded a partial response with progression-free survival of 8 months. Second-line lorlatinib in July 2022 led to progression after 1 month. Third-line albumin-bound paclitaxel (200 mg on days 1 and 8) + carboplatin (600 mg on day 1) for 2 cycles achieved stable disease. Fourth-line albumin-bound paclitaxel + carboplatin + tislelizumab for 3 cycles (October 2022 to January 2023) resulted in partial response. After progression in February 2023, fifth-line treatment included 14 cycles of local radiotherapy to the right skeletal bone and one cycle of oral anlotinib, discontinued due to intolerance. Sixth-line albumin-bound paclitaxel + carboplatin + tislelizumab for 1 cycle in March 2023 led to progression.

**Figure 2:**
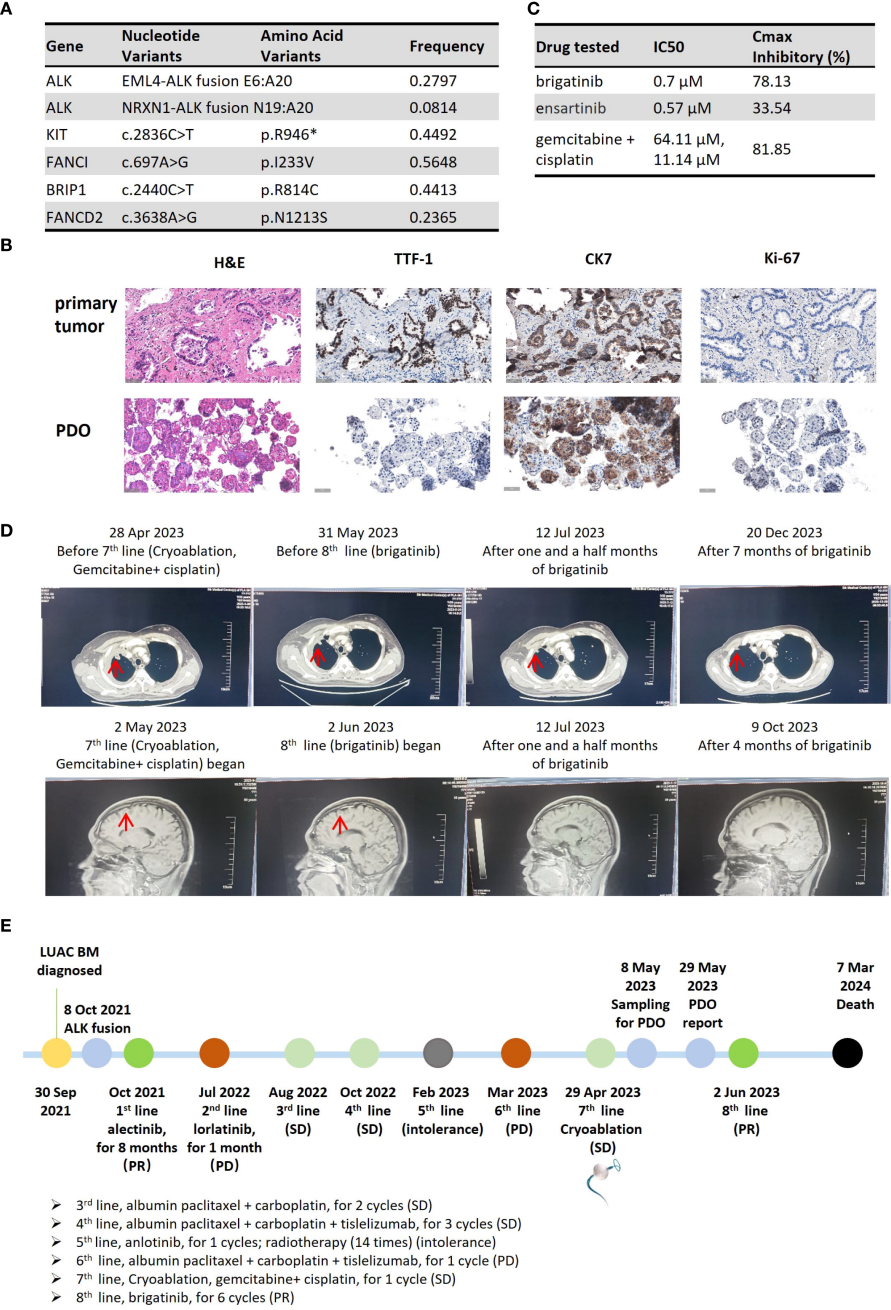
Clinical course of Case 2, a patient with dual *ALK*-fusion NSCLC. **(A)** Table of somatic mutations and fusions detected in the metastatic tumor tissue. **(B)** Histological comparison confirming the fidelity of the PDO to the metastatic pleural tumor. **(C)** Results of the PDO drug sensitivity assay. The table displays the IC_50_ and maximal inhibitory rate for each tested agent. (IC_50_ values represent the mean of technical replicates; standard deviations are not reported). **(D)** Serial imaging showing a PR after initiation of 8th-line therapy with brigatinib. **(E)** Timeline of the patient’s extensive clinical course, culminating in a PDO-guided therapy selection. (H&E, hematoxylin and eosin; IHC, immunohistochemistry; TTF-1, thyroid transcription factor-1; CK7, cytokeratin 7; PDO, patient-derived organoid; IC_50_, half-maximal inhibitory concentration).

On April 27, 2023, the patient was admitted to our hospital with imaging-confirmed progression. CT-guided percutaneous puncture of a right pleural metastatic lesion with combiknife cryoablation, digital subtraction angiography, bronchial artery embolization, and perfusion chemotherapy (gemcitabine 1.6 g + cisplatin 120 mg) was performed as seventh-line therapy. Three fresh samples from the May 8, 2023, puncture were sent for PDO culture and testing (Beijing Daxiang Biotech, China). Next-generation sequencing (NGS) of the metastases confirmed retention of both ALK fusions (**Figure 2A**).

PDO results on May 29, 2023, showed brigatinib with 78.13% inhibition (IC50: 0.7 μM) and ensartinib with 33.54% (IC50: 0.57 μM). Gemcitabine + cisplatin achieved 81.85% inhibition (IC50: 64.11 μM and 11.14 μM) (**Figure 2C**). Organoids preserved source tumor features, including CK7, Ki-67, and TTF-1 expression (**Figure 2B**). The report was issued 21 days post-collection.

On May 30, 2023, follow-up imaging showed stable disease after 1 month of gemcitabine + cisplatin. Although the treatment regimen achieved some disease control, the patient opted to try the oral brigatinib, which demonstrated the second-highest inhibition rate in the PDO testing and offered a more convenient administration route. Eighth-line brigatinib started on June 2, 2023, with partial response on July 10, 2023 (**Figure 2D**). Adverse events included rash and diarrhea. Disease remained stable until November 24, 2023 (5.8 months). The patient died from progression and multi-organ failure 9 months after initiating PDO-guided brigatinib. The treatment timeline is shown in **Figure 2E**.

## Discussion

PDO derivation and testing are integral to precision medicine in lung cancer, especially for alterations of unknown clinical significance (11–13). PDOs preserve the biological and genetic characteristics of parental tumors, reflecting changes in gene expression and metabolism induced by prior therapies (14, 15).

For newly diagnosed EGFR-mutated NSCLC with brain metastases, osimertinib-chemotherapy is often recommended, but patients may desire reduced burden during maintenance. In China, monthly hospital visits and biochemical monitoring for chemotherapy add time and cost. Many prefer oral osimertinib alone once stable. Here, PDO insensitivity to osimertinib monotherapy prevented premature de-escalation, emphasizing the need for informed regimen adjustments.

In Case 2, initial detection of ALK v3 fusion with novel NRXN1-ALK (N19:A20) led to progression after 8 months of alectinib and inefficacy of lorlatinib. Subsequent lines failed to control progression, with ALK fusions persisting in metastases. ALK v3 fusions are refractory; studies show median PFS of 34.9 months with alectinib versus 14.6 months with crizotinib (9, 16). Third-generation ALK inhibitors yield longer PFS than second-generation ones in brain metastases (17). Dual fusions may confer sensitivity or resistance to ALK inhibitors and chemotherapy (18–20). We hypothesize the novel fusion contributed to resistance, complicating treatment of brain and systemic metastases. PDO results indicated sensitivity to brigatinib and gemcitabine + cisplatin, and resistance to ensartinib. This aligned with stable disease on gemcitabine + cisplatin and subsequent brigatinib benefit. Certain gatekeep mutations may emerge after treatment with alectinib. However, our study did not perform targeted sequencing on the PDO samples to identify specific EML4-ALK fusion variants or co-occurring mutations. As highlighted by Lin et al. (21), brigatinib has demonstrated activity in patients with alectinib-refractory ALK-positive NSCLC. This study identified several potential mutations that can arise after alectinib treatment, which may influence the response to subsequent therapies such as brigatinib.

Case reports show PDOs guiding treatment in LRRTM4-ALK fusions (13) and EGFR-negative NSCLC (22). PDOs can aid diagnosis and therapy even from unknown primaries, metastases, lymph nodes, or ascites (23, 24), advancing precision oncology.

## Limitations

This study has several limitations. Firstly, as a report of only two cases, its findings lack statistical power and cannot be generalized. The PDO models, while useful for drug screening, lack an immune microenvironment and other systemic factors, representing an inherent simplification of *in vivo* tumor biology. Furthermore, we did not report the overall success rate of PDO establishment, which is a key metric for assessing feasibility. Based on published literature, the success rate for establishing NSCLC PDOs can vary, typically ranging from 70% to 88%, depending on factors such as sample type, tumor cellularity, and culture techniques (25, 26). Secondly, these organoids were not used for downstream mechanistic studies to formally explore the basis for drug resistance or the functional role of the novel ALK fusion. Lastly, we are working to shorten the 21-day turnaround time for testing to better align with urgent clinical decision-making timelines.

## Conclusion

These two case reports illustrate the utility of PDO-based drug sensitivity assays in newly diagnosed and multi-line progressed patients with lung cancer brain metastases. They encourage clinicians to leverage PDOs for selecting personalized regimens, especially when genomic profiling offers multiple options.

## Data Availability

The original contributions presented in the study are included in the article/Supplementary Material. Further inquiries can be directed to the corresponding author.
